# Programmable droplets: Leveraging digitally-responsive flow fields to actively tune liquid morphologies

**DOI:** 10.1371/journal.pone.0264141

**Published:** 2022-03-23

**Authors:** Raphael Kay, Charlie W. Katrycz, Ethan J. Heimlich, Benjamin D. Hatton

**Affiliations:** 1 Department of Mechanical and Industrial Engineering, University of Toronto, Toronto, Ontario, Canada; 2 Department of Materials Science and Engineering, University of Toronto, Toronto, Ontario, Canada; 3 Department of Statistical Sciences, University of Toronto, Toronto, Ontario, Canada; Tianjin University, CHINA

## Abstract

Stimulus-responsive materials enable programmable and adaptive behaviors. Typical solid-phase systems can only achieve small deformations for applications where shape transformations are beneficial or required. Liquids, in contrast, can self-assemble and achieve very high strains in a multifluid environment. Here we report liquid droplet formation by tuning flow potential within a confined fluidic cell. We digitally inject small volumes of liquid-pigment into an otherwise-transparent liquid layer, generating macroscopic droplet assembly over large areas constrained between closely-spaced plates. Droplet morphology is actively controlled by modulating outlet conditions to tune flow fields. Pattern stability is maintained through control over injection rate, interfacial viscosity difference, and interfacial surface tension. We demonstrate time-dependent droplet formation and migration to achieve spatially-tunable optical properties. Applied as a multi-cell array, we imagine this liquid mechanism will enable scalable pattern dynamics for active shading and visual display technologies.

## Introduction

Smart materials, which are typically characterized by an integrated capacity to sense, control, and actuate in response to an applied stimulus, enable trainable and reactive behaviors at the material scale [1–3]. Conventionally, solid-phase systems achieve volumetric changes and/or shape deformations, caused by microscopic changes of the lattice structure, when subjected to thermal [4, 5], optical [6, 7], hygroscopic [8–12], or electric stimuli [13, 14]. In this article, we instead propose a liquid-phase smart material system that achieves pigment self-assembly under the influence of tunable potential liquid flow. We manipulate various boundary pressures within a confined fluidic cell, enabling flow-responsive liquid deformation in a reversible and repeatable manner.

Intermolecular forces are much weaker in liquids than solids. As a result, liquids easily flow and can be simply manipulated. Pressure-induced liquid flow has been leveraged to achieved dynamic thermal [15–19] and optical [15] properties in materials. Infrared-absorbing liquids, for instance, can be pumped through a window-embedded vasculature, enabling cooling effects along the building interface [15]. Liquids stored within a deformable bladder can also be displaced to proportionally measure pressure, providing a microfluidic mechanism for active sensing and display [20]. Moreover, fluids can carry nano- and microscopic particles that enable more sophisticated responses to applied stimuli. For example, colloidal suspensions of ferro- or ferrimagnetic particles can form complex ferrofluidic morphologies in applied magnetic fields [21–24].

Capillary interactions, which occur at the length-scale of a fluidic droplet, can also dictate sophisticated and choreographed liquid movements. Droplets can be manipulated on solid surfaces by topographical [25–27], chemical [25, 28, 29], or electrical [30, 31] patterning. Through designed functional gradients, liquids can be made to overcome resistant forces, enabling directed droplet transport in a specified direction. One famous study demonstrated how a gradient in surface free energy (hydrophobicity) could cause a water droplet to run uphill [27].

Liquid morphologies can also be controlled within confined multifluid environments. In particular, fluid dynamics are commonly investigated in Hele-Shaw cells–comprising a thin liquid layer constrained between two closely-spaced rigid plates. Viscosity-driven interfacial instabilities, for instance, are widely characterized within Hele-Shaw systems, where a less viscous fluid is introduced into a more viscous fluid under pressure [32]. Known as the Saffman-Taylor (viscous fingering) instability, liquid patterns emerge due to small perturbations in their flow. These perturbations can propagate along the multifluid interface, forming fractal-like branching features [33–36].

In this work, we present a new mechanism for tuning liquid droplet morphology: programmable potential flow. We demonstrate control over droplet shape and position by switching the pressure condition (i.e., either open to atmosphere or closed) of a series of active valves along a Hele-Shaw cell boundary. This control over pressure allows us to manipulate local flow fields, enabling time-dependent droplet shape-change and migration. By tuning the interfacial properties between droplet and host liquid phases, we ensure stable interfacial growth. And by introducing precise concentrations of pigment particles within these liquid droplets, we can use flow fields to tune patterns of optical transmission and absorption across a cell. This liquid smart material can be demonstrated from the microscopic to macroscopic length-scale. And we speculate that this generalizable methodology for switchable droplet morphology and migration will enable new possibilities for dynamic display, communication, camouflage, and shading.

## Materials and methods

**Fig 1:**
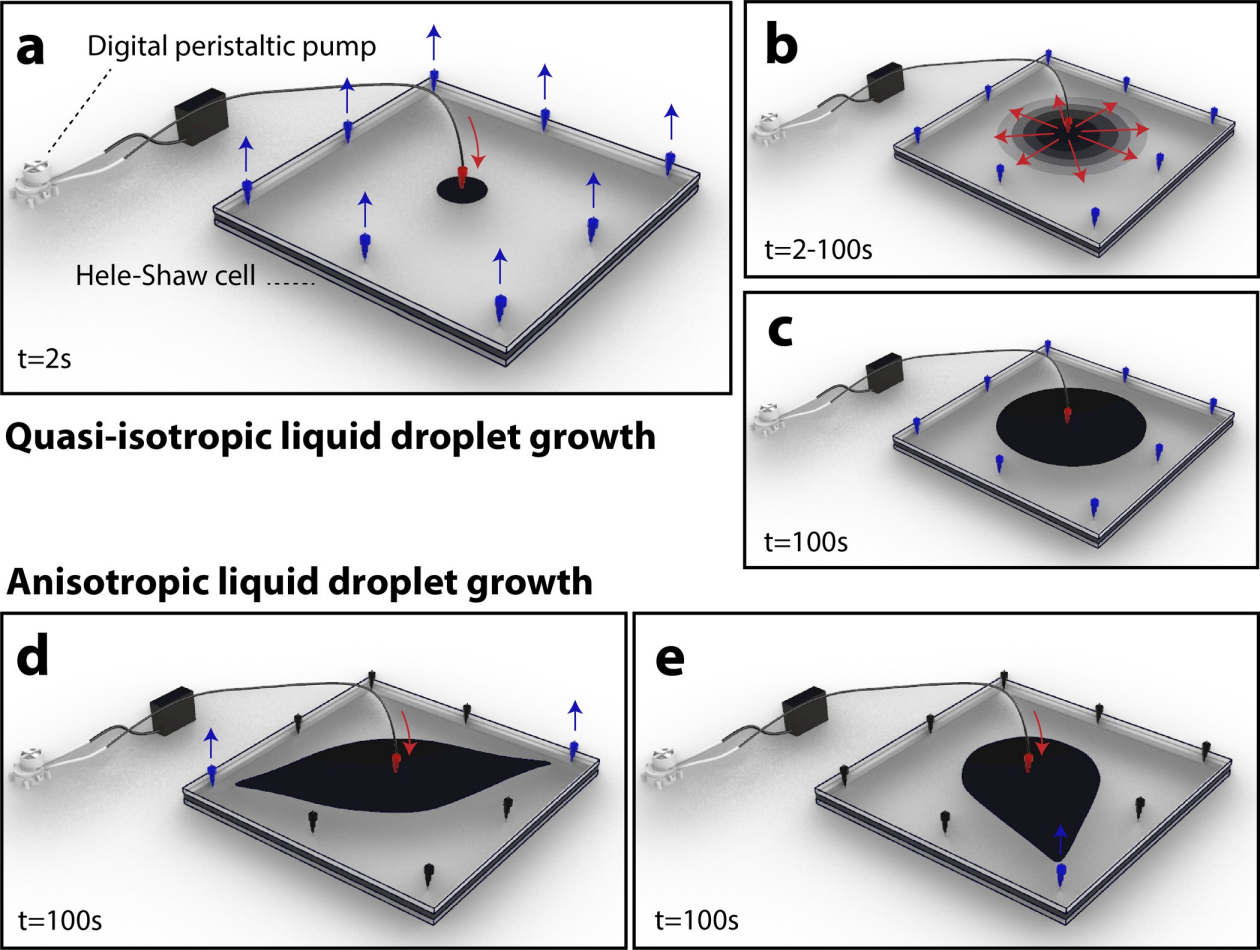
Working principle of active droplet formation. (a-c) Digitally-driven quasi-isotropic pigment injection within Hele-Shaw fluid cell. (d-e) Anisotropic droplet formation over time achieved through dynamic control of flow field.

We fabricated a modified Hele-Shaw cell, constrained by two rigid PMMA plates (30x30x0.6 cm^3^). Plates were separated and sealed at the edges using a 2-mm-thick double-sided elastomer adhesive (3M). We machined one central injection port at the center of, and eight outlet ports around the boundary of, one of the two PMMA plates (Fig 1A). We connected the central inlet using tubing to a bi-directional, NE-1010 digital syringe pump, and we connected switchable valves to each of the eight outlet ports. We opened all boundary valves, and filled the Hele-Shaw cell with a clear castor oil (288 cP, 0.88 g/cm^3^).

To generate a pigment-phase droplet, we introduced an aqueous suspension of carbon black (288 cP, 1.09 g/cm^3^) into the castor oil already present within the Hele-Shaw cell. Carbon particles were sonicated to decrease particle clustering and improve suspension longevity. The pigment phase represented a suspension of carbon black at a concentration of 0.2 g/50 mL of a glycerol-water mixture (note that glycerol and water are miscible in all proportions, and can mix with no significant change in relative molar volumes [37]). The viscosity and density of the glycerol-water mixture were calculated using the four-parameter correlation of temperature-dependence on aqueous glycerol solution viscosities, presented by Chen and Pearlstein [38]. Calculated viscosity values were then compared to those generated experimentally by Segur and Oberstar [39]. We tested the viscosity effect of the carbon black by comparing timed capillary flow through a Cannon-Fenske capillary viscometer for both a sample of distilled water and a distilled water suspension of carbon, and confirmed a negligible difference (< 1%). The viscosity of the castor oil phase was also calculated experimentally using a Cannon-Fenske capillary viscometer, and interfacial surface tension between the pigment and oil phases was estimated from the literature [37]. Finally, optical spectra for both the pigment and oil phase liquids, were measured with a PerkinElmer Lambda 1050 Spectrometer (three-detector module).

Droplets were introduced within Hele-Shaw cell (25 mL/min), displacing the host castor oil towards the boundary outlets. Outlet valves were switched for each potential flow configuration, and open valves were connected to drainage tubing, collecting in a shared reservoir open to the atmosphere. Droplets were then reversibly collapsed by reversing the flow on the syringe pump.

We assumed Darcy flow in the Hele-Shaw cell, where instantaneous flow rate is proportional to the pressure gradient, *q*∝∇*P*. This form of flow is mathematically equivalent to an electrostatic problem where field strength is proportional to the gradient of electrostatic potential, *E*∝∇*V*. Steady-state, potential driven flow, for each outlet configuration, was approximated by modifying a Laplacian solver originally written for electrostatics (MATLAB code adapted from [40]).

## Results

### Reversible quasi-isotropic droplet injection

**Fig 2:**
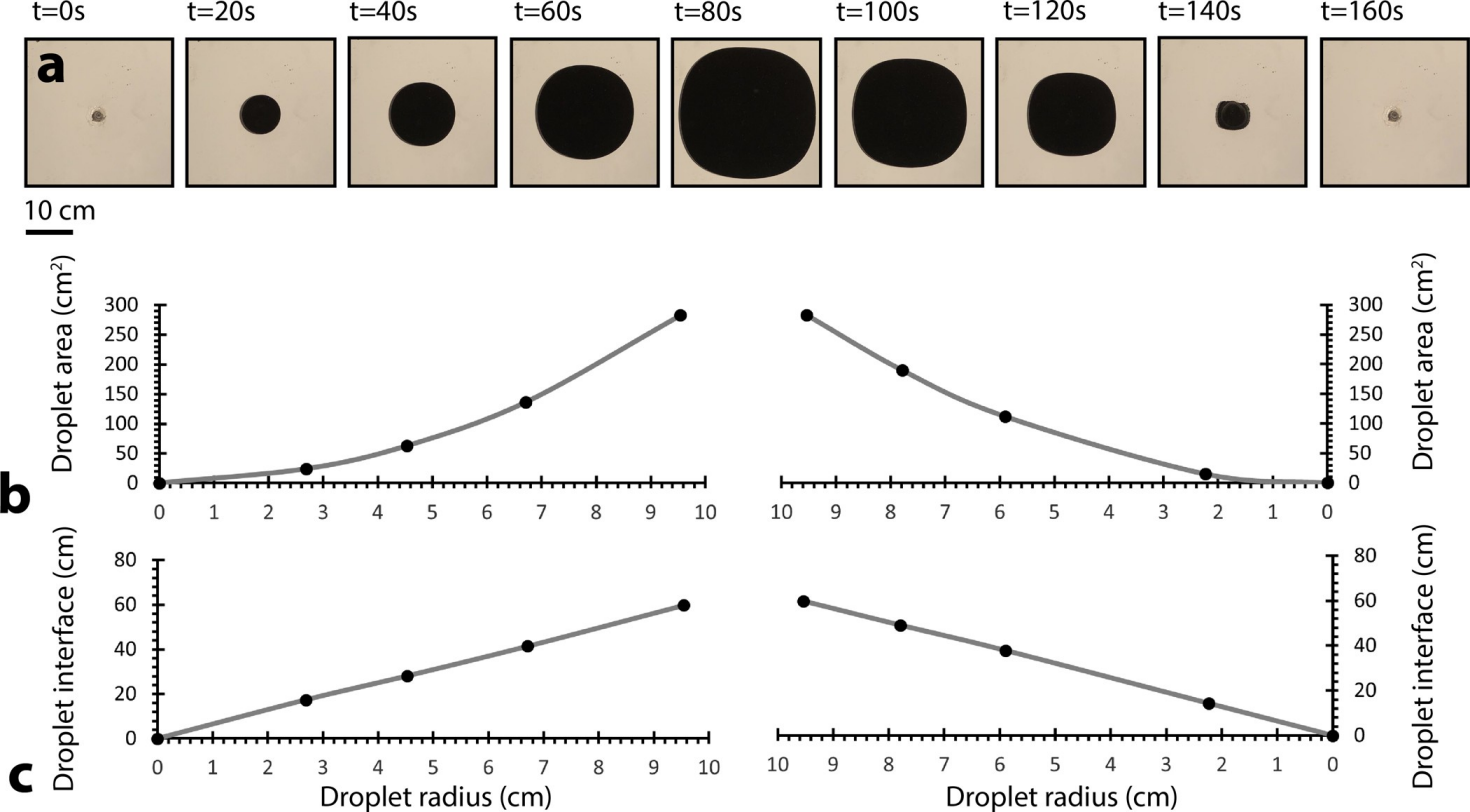
(a) Reversible pigment droplet dispersal and retraction under quasi-isotropic flow field. Images show fifth dispersal/retraction sequence of fifty similar sequences. (b) Droplet area as a function of droplet radius, where droplet area is proportional to dispersed volume, for reversible droplet dispersal and retraction sequence. (c) Droplet interface (perimeter) as a function of droplet radius for reversible droplet dispersal and retraction sequence.

We injected viscous, aqueous phase, pigments into a transparent oil phase contained within a Hele-Shaw cell. Each of the eight outlet boundaries of the cell was opened to atmospheric pressure (Fig 2A), and pigment was injected at a rate of 25 mL/min. Pigment droplets grew radially under a quasi-isotropic potential field (Fig 2A–2C). Consistent with a circle, droplet area increased with the square of its radius (Fig 2B), and droplet perimeter increased linearly with its radius (Fig 2C).

Droplet growth was repeatable across multiple cycles (n>50), but exhibited a small amount of hysteresis (Fig 2). We speculate that plate elasticity contributed to this growth direction asymmetry. During droplet growth, at positive pressure, the spacing between plates likely expanded, aiding radial droplet morphology. During droplet collapse, at negative pressure, the spacing between plates likely decreased, contributing to a thinning phenomenon and loss of radial symmetry (Fig 2A, t = 140s).

### Anisotropic droplet injection

**Fig 3:**
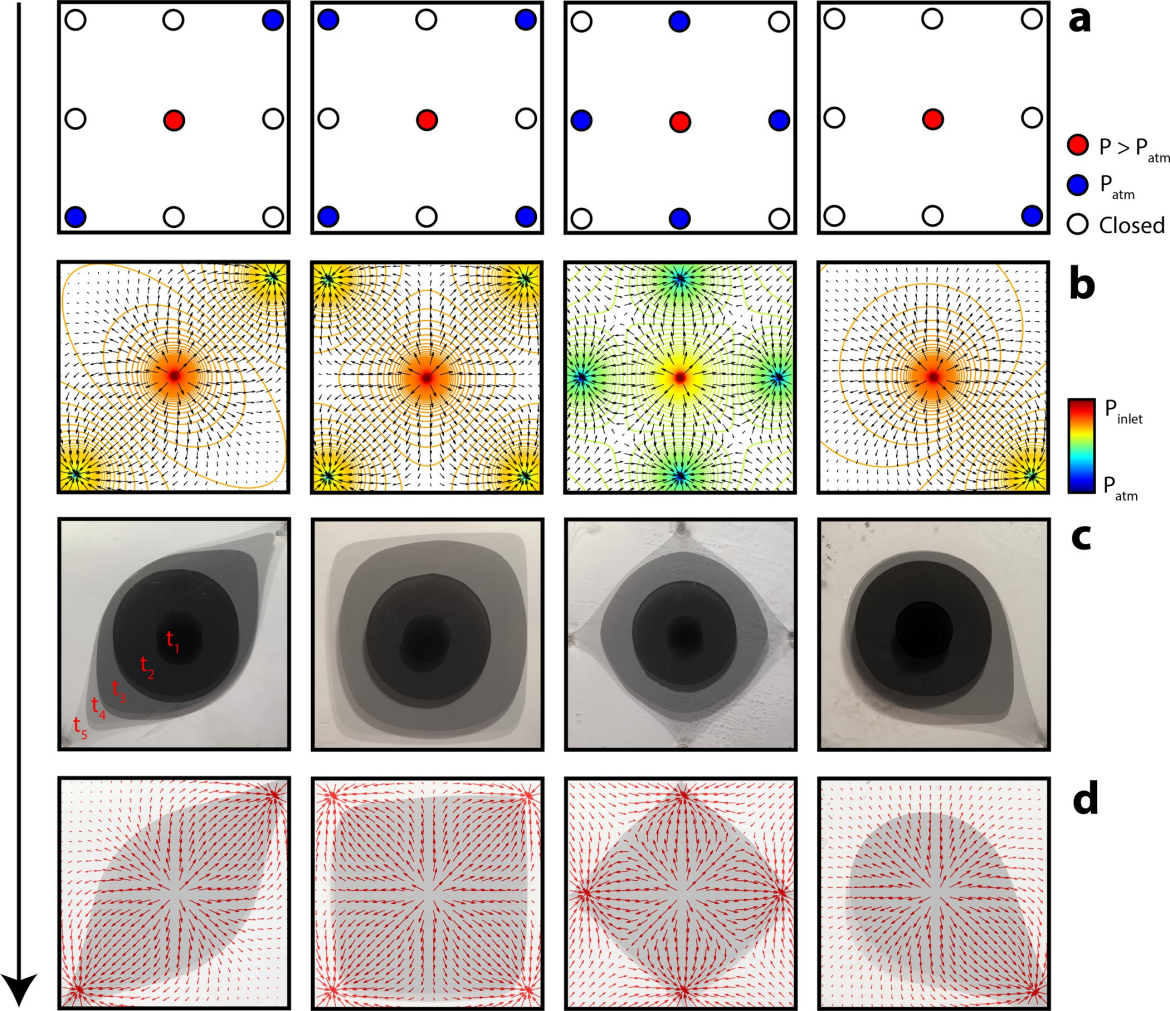
Pigment dispersal across variable anisotropic flow fields. (a) Four Hele-Shaw cell states with spatially-varied outlet boundary conditions. (b) Estimated (idealized) steady-state potential flow field from cell configurations in a. (c) Overlaid time-series images demonstrating anisotropic droplet growth over time. (d) Final droplet configuration from c with overlaid gradient vector field from b.

**Fig 4:**
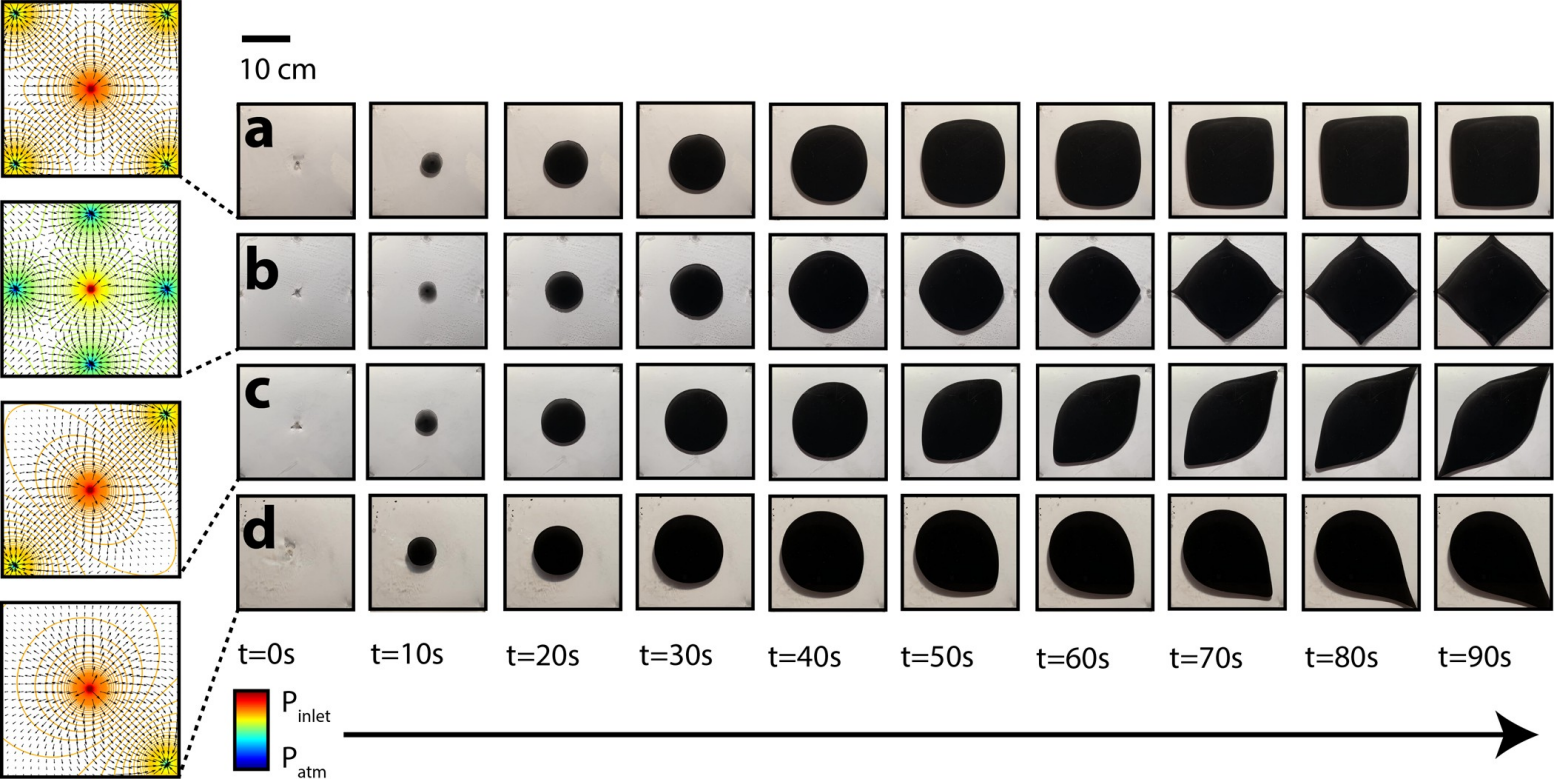
Time-dependent droplet growth under four unique potential flow fields (a-d).

We again injected viscous, aqueous phase, pigments into a transparent oil phase contained within a Hele-Shaw cell. This time, however, outlet valves along the cell boundary were selectively closed (Fig 3A), generating anisotropic potential flow across the cell (estimated in Fig 3B). Pigment was injected at a rate of 25 mL/min, and droplets grew under an anisotropic field (Fig 3C). Droplets developed initially with a radial morphology (Figs 3C, **4**A–**4**D), where anisotropic potential flow was not sufficient to overcome droplet surface energy and host fluid viscosity. After 30 seconds (12.5 mL of pigment injected), however, pigment droplets began to deform according to the potential flow gradient estimated in Fig 3B (Figs 3D, **4**A–**4**D).

At equilibrium, pigment droplets expanding within a confined Hele-Shaw cell will assume a circular shape, to minimize surface energy. Under a hydrodynamic flow field, however, viscous stresses (proportional to the flow shear rate, and viscosity) will act on the droplet to cause it to deform. Accordingly, there is a competition between forces acting on the droplet: the viscous forces to deform, and the capillary forces (through surface tension) acting to return the droplet to an equilibrium shape.

The droplet deformation can be explained by assuming Darcy flow with the Hele-Shaw cell, with source and sink boundary conditions. For flows obeying Darcy’s law, instantaneous flow rate is proportional to the pressure gradient, *q*∝∇*P*. In total, we injected 20 mL of pigment into the Hele-Shaw cell, and observed continuous droplet deformation (Figs 3C, 4A–4D). Idealized, steady-state, potential-driven flow was estimated for various outlet configurations in Fig 3B.

### Time-dependent droplet morphology growth

**Fig 5:**
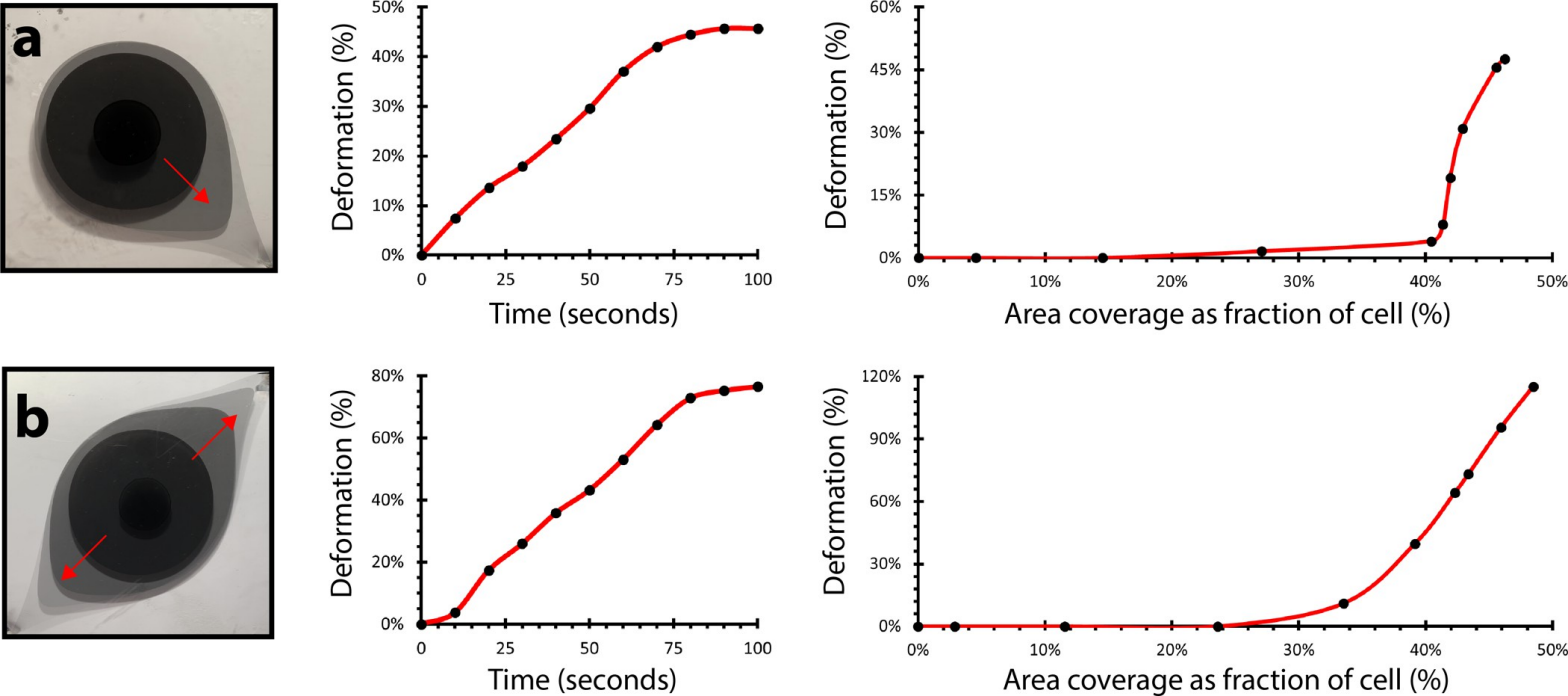
Non-normalized (a) single-outlet and (b) multioutlet droplet deformation as a function of time (left graphs) and area-normalized droplet deformation as a function of fractional area coverage (right graphs), proportional to dispersed pigment volume (flow rate = 25 mL/min).

Droplet morphology changed with time under anisotropic flow fields (Fig 4A–4D). Active injection in all cases lasted approximately 48 seconds (20 mL, at 25 mL/min), while deformation continued until the active droplet reached a boundary outlet (approximately 100 seconds). We first characterized droplet growth and deformation over time for both single-outlet (Fig 5A) and double-outlet (Fig 5B) cell configurations (Fig 4D and 4C, respectively). The left graphs in Fig 5 describe droplet deformation as a function of time. Droplet deformation, defined here as the change in droplet length in the direction of greatest possible deformation (i.e., towards an outlet) moves linearly with time. The right graphs in Fig 5 describe area-normalized droplet deformation as a function of pigment coverage. Normalized droplet deformation, defined here as the relative difference between the droplet length in the direction of greatest potential and the droplet length in the direction perpendicular to the direction of greatest potential, increases later in the growth process, and rapidly (> 30%, right graphs, Fig 5). In the single-outlet case, we see that relative droplet deformation almost exclusively occurs after the injection sequence has finished, as the full 50% relative deformation is achieved with only a 6% change in pigment area coverage (40%-46%) (Fig 5A, right graph). In the double-outlet case, relative droplet deformation occurs exclusively in the final half of pigment injection (between 25–49% area coverage) (Fig 5B, right graph). In both these injection sequences, accelerated flow along the droplet tip closest to the outlet was observed, consistent with expected flow outcome from the estimated gradient fields (Fig 3B).

**Fig 6:**
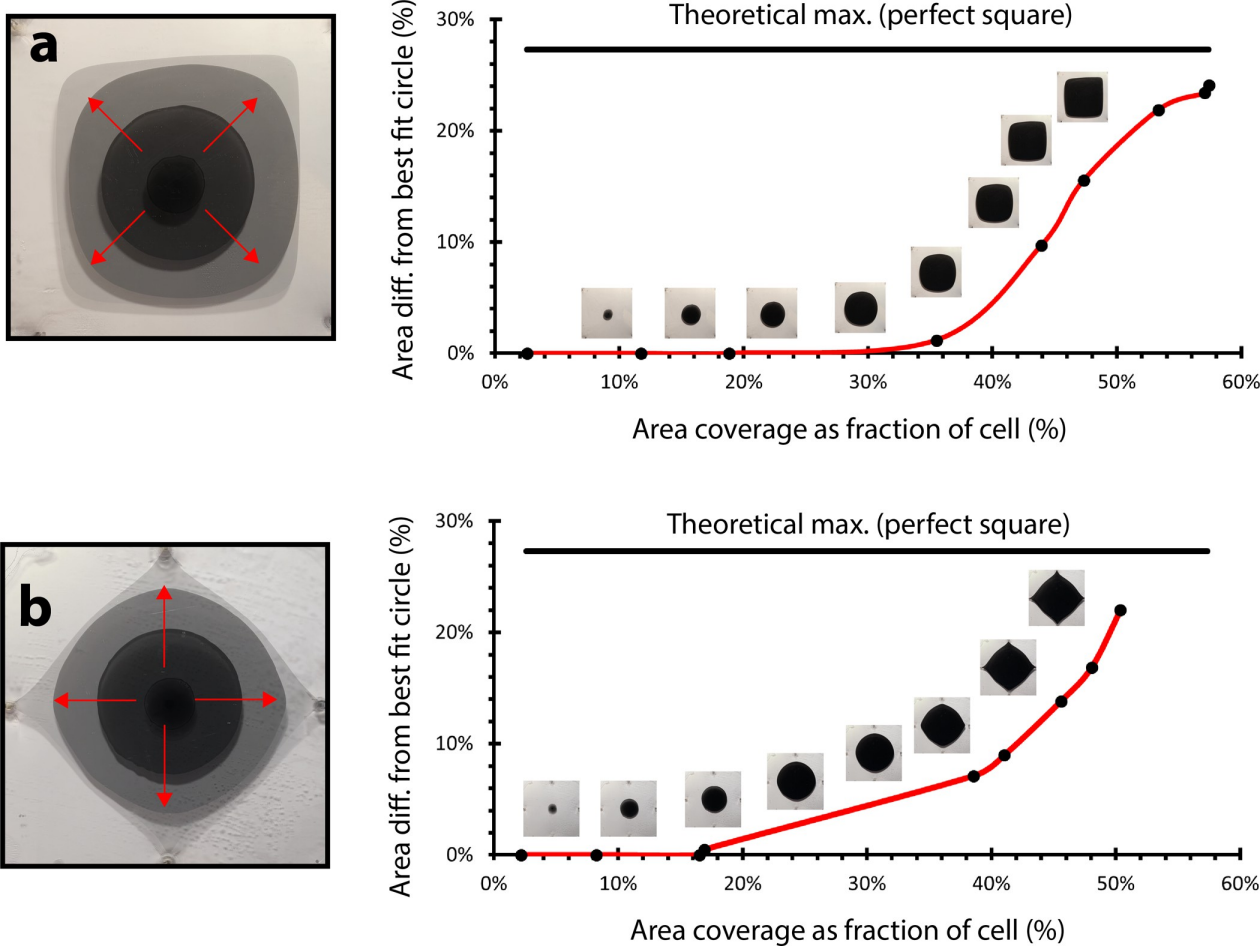
Circular droplets become more square-like over time. Quad-outlet droplet deformation as a function of fractional area coverage, proportional to dispersed pigment volume (flow rate = 25 mL/min), for both (a) corner outlet configuration and (b) edge midpoint outlet configuration.

For injection sequences corresponding to quad-outlet configurations (Fig 4A and 4B), we characterized time-dependent pigment morphology as droplets grew from circles to approach the shape of a square (Fig 6A and 6B, respectively). For a corner-diagonal distribution of boundary outlets (outlet at each corner), droplets changed in morphology from the theoretical minimum area difference from the best fit circle (0%, as the droplets were near-circles) to approach the theoretical maximum area difference from the best fit circle (28%, as the droplets became squares) (Fig 6A). As flow potential was distributed across all corners of the cell, and outlets were positioned at a maximum distance from the central injection site ($d=\sqrt{2}r$), where *r* is half a boundary length of the square cell, the fixed-volume (20 mL) pigment droplet did not reach any of the four outlet boundaries. Accordingly, a near-square morphology was reached prior to complete injection (Fig 6A, decreasing slope between 55–59% area coverage).

For an edge-midpoint distribution of boundary outlets (outlet at each edge midpoint), droplet deformation also increased from the theoretical minimum area difference from the best fit circle (0%) to approach the theoretical maximum area difference from the best fit circle (28%, square) (Fig 6B). While flow potential was again across multiple regions of the cell, outlets were positioned at a distance 29% closer ($\{\sqrt{2}r-r\}/\sqrt{2}r$) from the central injection site (*d* = *r*), enabling the pigment droplet to reach outlet boundaries. Accordingly, unlike the injection sequence demonstrated in Fig 6A, the rate of pigment deformation increased continuously across the entire injection sequence (Fig 6B, increasing slope).

### Time-dependent droplet migration

**Fig 7:**
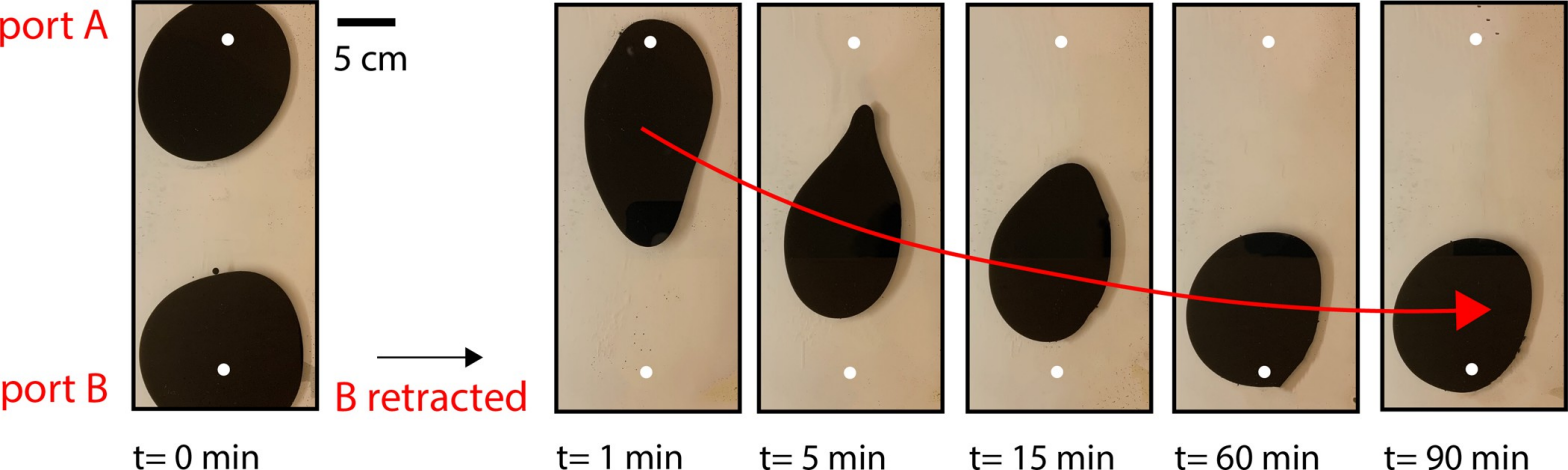
Multiport time-dependent droplet migration due to a hydrodynamic potential.

Beyond droplet morphology tunability, we also demonstrated the capacity for complete droplet migration. More than just controlling shape, the capability to choreograph the movement of a droplet, and, in aggregate, a sequence of droplets, might enable more versatile and efficient display systems. Far less material and energy are required to move a pigment droplet across a cell than to orchestrate several sequential droplet growth and retraction sequences that can only simulate droplet movements in low resolution. We fabricated a multiport Hele-Shaw cell, with two injection ports situated at opposite cell regions (Fig 7), and a series of evenly-distributed outlet drains (open to atmospheric pressure) to enable drainage proportional to injected pigment volume. We injected two pigment droplets simultaneously into the cell, and let the fluid within the cell reach a pressure-equilibrium. We then closed all outlet drains, and retracted one of the two pigment droplets completely. Through this retraction sequence, we generated a pressure-driven flow field from the injection port with the remaining droplet to the injection port with the recently-retracted droplet. Over 90 minutes, we observed complete droplet migration, until the fluid cell once again reached a pressure-equilibrium (Fig 7, t = 1–90 min).

### Multiport patterning

**Fig 8:**
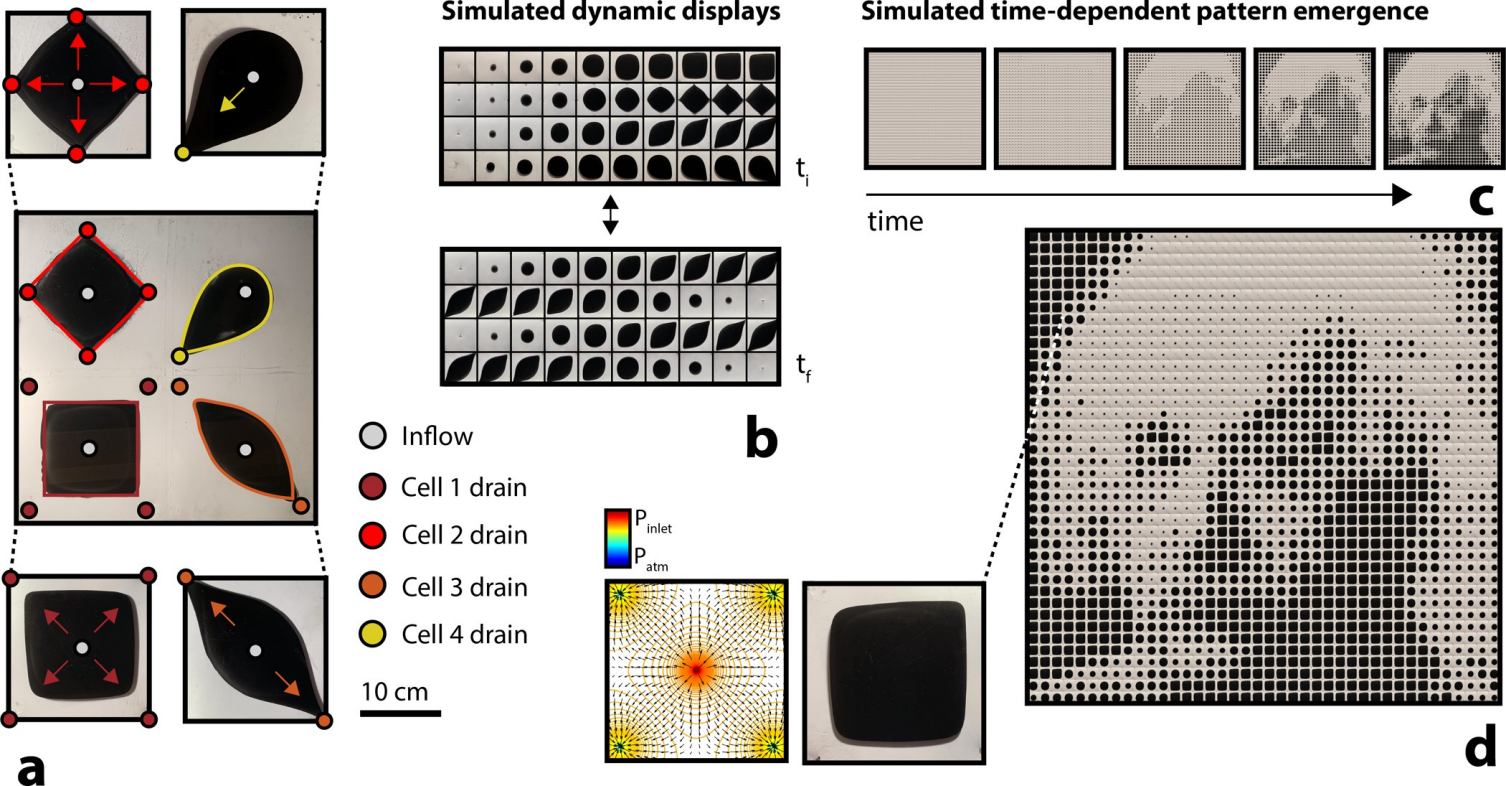
Fabricated and simulated multipixel visual displays. (a) Image of a built quad-cell display, where each array element demonstrates a unique flow field and pigment droplet morphology (t = 100 s after injection). (b) Examples of switchable digitally-generated multicell displays (unbuilt). (c) Simulated pattern emergence to achieve a predefined multicell visual array (d). Static flow field displayed next to corresponding pigment cell. Assuming a cell length of 20 cm, the visualized 40x40 cell display could stretch 8x8 m^2^.

We fabricated a multicell display, designed as a 2x2 array of the Hele-Shaw system illustrated in Fig 1. We demonstrated differential pattern dynamics within the array, generating independent, digitally-switchable responses across each Hele-Shaw cell (Fig 8A). We imagine the upscaling of this array, and generated digital multicell pattern arrangements to visualize more complex, coordinated pigment droplet responses (Fig 8B). To emphasize the potential of this system as a large-area adaptive display, we developed a computational program to read binary digital images and map, based on localized greyscale values, appropriate experimental pattern results. The program was developed to take any two-dimensional multi-pixel image, convert each colored pixel into a grey-scale value between 0–255, and then lower the resolution of the image by averaging the values of neighboring pixels to produce a coarse pixel resolution. Next, the program was designed to replace each individual pixel with an experimental image of our droplet growth, to simulate fluidic display. In particular, larger (more advanced) pigment droplets were proportionally mapped to smaller (darker) greyscale pixel values (Fig 8C–8D). In our case, we sorted pixel values into five buckets of increasing grey scale values, and we matched pixels within each of the buckets with five experimental captures of increasingly-sized pigment droplets. We extrapolated time-dependent pattern emergence (Fig 8C) to achieve coarse-resolution grey-scale imagery (e.g., an Albert Einstein portrait; Fig 8D). In the first image in Fig 8C, after no elapsed time, all pixel values were mapped to images of the experimental droplet injection after no elapsed time, when the bubble had not yet emerged. In sequential images following the first, pixel values within increasing ‘buckets’ were mapped to experimental images of increasingly-larger pigment droplets–up until the fifth image, where pixels sorted within each of the five available buckets were mapped to their proportional experimental image.

## Discussion

### Differential optical programmability

**Fig 9:**
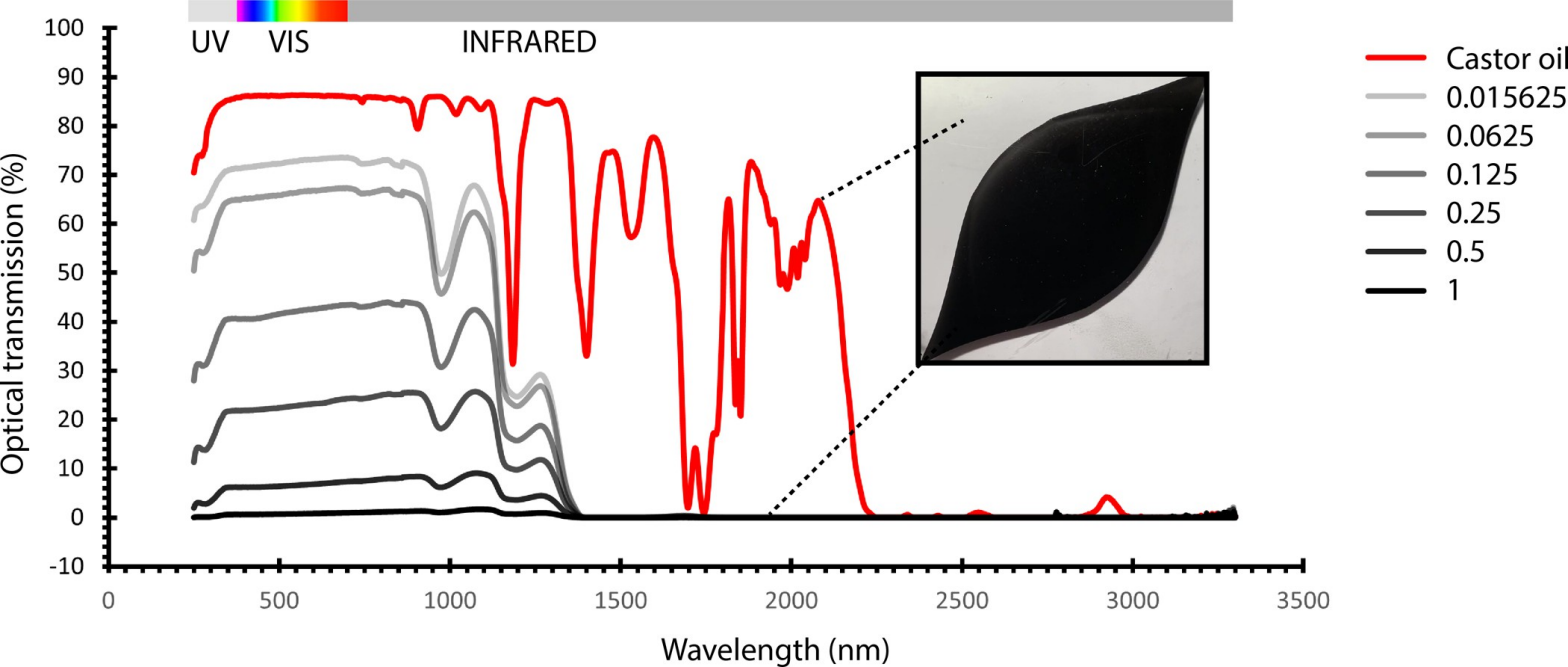
**Differential visible and near-infrared light transmission between the aqueous pigment phase (grey curves) and oil phase (red curve) for a 3 mm optical path length.** Grey curves represent varied concentrations of aqueous carbon black suspensions, where values describe concentration in g of C per 50 mL of H_2_O.

We have demonstrated a methodology for generating dynamic pigment morphologies within confined fluidic cells. Active droplet growth and migration enables spatially-tunable optical properties, where precise optical absorption and transmission can be programmed both locally, by actively controlling flow potential and growth, and globally, by scaling pixelated fluid control across large-area arrays. We characterized optical transmission in both the visible and near-infrared spectrum for the transparent host phase (castor oil), and the aqueous pigment phase (glycerol carbon suspension) for an optical path-length of 3 mm within our cell (Fig 9). We generated active spatiotemporal switching between host and pigment liquid phase optical properties, which can induce changes to both visible light transmittance and solar heat gain (Fig 9). We also demonstrated significant differences in optical transmission within the visible spectrum for a range of aqueous-phase carbon suspension concentrations (Fig 9). Fluids have easily-tunable absorption properties [41, 42], and fluid flow represents a novel and scalable mechanism for large-area optical control.

### Reversibility limitations

Large fluid deformations introduce reversibility constraints. High-viscosity, high-surface-tension liquids decrease undesired fluid mixing (droplet ‘island’ formation), however, above an estimated capillary length (~20 cm), pigment droplets are still susceptible to splitting (separation of droplets) during retraction. Additionally, droplet growth becomes irreversible once the pigment interface extends into an outlet boundary. With this constraint, Hele-Shaw cell length-scale also dictates the maximum pigment droplet size. In our work, within a 30x30x0.2 cm^3^ Hele-Shaw cell, we report reversible droplet morphology for droplets up to 22 cm in length (73% the length of the cell).

### Future work

We have introduced and demonstrated a simple material mechanism for transforming the shape of a two-dimensional pigment droplet within a confined cell. We speculate that the development of multicell arrays, coupled with centralized digital control over valve switching and pigment injection, will enable more sophisticated morphological outputs, for light regulation and surface display applications. We propose increasing the resolution of valve control by increasing the density of outlets (i.e., the density of control), not only along cell edges, but within the cell region. Additional characterization is required to understand the time-dependent flow and hydrodynamic potential that underlies droplet migration. We propose the development of a larger multiport Hele-Shaw cell, within which more sophisticated droplet movements, fusions, and separations, can be digitally-choreographed and performed. Ultimately, the work described here demonstrates a proof-of-concept. More advanced mechanical techniques concerned with integrating pigment and drain storage, as well as electronic valve, driver, and pump equipment, are required to achieve continuous, reversible, high-resolution pigment display.

## Supporting information

S1 Fig Electronic configuration to operate 8-cell system. Eight digital 12 V DC peristaltic pumps connected to Arduino controller and pump motor drivers. Wires on the top left of the image connect to a manual keypad, which can be programmed to receive user inputs.
(DOCX)
