# Supplementary material for: Programmable droplets: Leveraging digitally-responsive flow fields to actively tune liquid morphologies
Source: PLoS One. 2022 Mar 23;17(3):e0264141. doi: 10.1371/journal.pone.0264141 (PMC8942257; doi:10.1371/journal.pone.0264141)
Supplement: S1 Fig — Eight digital 12 V DC peristaltic pumps connected to Arduino controller and pump motor drivers. Wires on the top left of the image connect to a manual keypad, which can be programmed to receive user inputs. (DOCX) [file pone.0264141.s001.docx]

**Supporting Information**


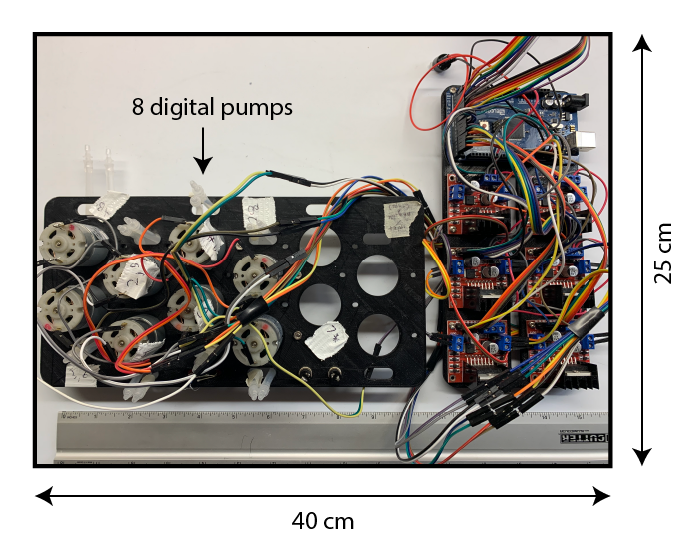


**S1 Fig. Electronic configuration to operate 8-cell system.** Eight digital 12 V DC peristaltic pumps connected to Arduino controller and pump motor drivers. Wires on the top left of the image connect to a manual keypad, which can be programmed to receive user inputs.
